# A simplified hydroxyurea dosing approach for paediatric sickle cell anaemia in nigeria: addressing emergency care burden and adherence barriers in low-resource settings

**DOI:** 10.1186/s12887-025-05834-y

**Published:** 2025-07-02

**Authors:** Chisom Nri-Ezedi, Nwanneka Ugwu, Arinze Ulasi, Thomas Obiajulu Ulasi

**Affiliations:** 1 Department of Paediatrics, Nnamdi Azikiwe University, Awka, Anambra Nigeria; 2 Department of Paediatrics, Nnamdi Azikiwe University Teaching Hospital, Nnewi, Anambra Nigeria

**Keywords:** Sickle cell anaemia, Hydroxyurea, Treatment adherence, Simplified dosing, Nigeria

## Abstract

**Background:**

Sickle cell anaemia (SCA) is highly prevalent in sub-Saharan Africa and is characterized by frequent vaso-occlusive crises and other severe complications. Hydroxyurea has proven effective in reducing SCA complications by increasing foetal haemoglobin, but its use in low- and middle-income countries (LMICs) remains limited due to cost, need for regular laboratory monitoring, and concerns about safety. This study evaluates a simplified hydroxyurea dosing regimen as a practical approach to reduce emergency room visits and improve treatment adherence in paediatric SCA within a resource-limited setting.

**Methods:**

We conducted a prospective open-label cohort study over two years (January 2022– January 2024) at a tertiary hospital in South East Nigeria. One hundred children aged 1–18 years with confirmed SCA (HbSS genotype) were started on a uniform dose of hydroxyurea (20 mg/kg/day, capped at 500 mg/day). Patients were followed monthly, and those experiencing “breakthrough vaso-occlusive crises” had their dose increased to 25 mg/kg/day (or from 500 mg to 750 mg for those at the capped dose). The primary outcome was the frequency of SCA-related emergency room visits. Secondary outcomes included treatment adherence (assessed via caregiver report of missed doses) and factors associated with adherence. Statistical analyses (t-tests, Mann–Whitney U, chi-square) were performed to compare clinical and demographic variables between adherence groups, with a significance threshold of *p* < 0.05.

**Results:**

The simplified hydroxyurea regimen dramatically reduced emergency room visits. Only one patient (1% of the cohort) required an emergency visit for a vaso-occlusive crisis during the two-year period, and this patient’s dose was successfully escalated to 750 mg/day with no further crises. Overall, treatment adherence was high: 84.2% reported consistent adherence to daily hydroxyurea. Younger children demonstrated better adherence than older children (mean age of adherent patients 8.5 ± 5.3 years vs. 11.6 ± 4.2 years for non-adherent, *p* = 0.027). Patients who were non-adherent tended to have older fathers (median paternal age 49 years vs. 44 years in adherent patients, *p* = 0.02). Other factors—sex, socio-economic status (social class), maternal age, weight, and baseline health status—showed no significant association with adherence (all *p* > 0.2). No severe adverse effects were observed, and the dosing approach was well-tolerated without routine laboratory monitoring.

**Conclusion:**

A simplified hydroxyurea dosing strategy appears to be a safe, effective, and feasible strategy for managing paediatric SCA in a resource-limited setting. This approach resulted in a substantial reduction in vaso-occlusive crises requiring emergency care and high levels of treatment adherence. These findings suggest that simplified dosing protocols could be a valuable component of broader SCA care strategies in settings such as Nigeria. Validation through larger or comparative studies is encouraged, and we recommend that healthcare policy-makers consider piloting and supporting similar models as part of national sickle cell control efforts.

**Supplementary Information:**

The online version contains supplementary material available at 10.1186/s12887-025-05834-y.

## Introduction

Sickle cell anaemia (SCA) is a hereditary haematological disorder predominantly affecting individuals of African descent, with significant global health implications [1]. It is characterized by the production of sickle-shaped erythrocytes, leading to recurrent vaso-occlusive crises and complications such as acute chest syndrome, stroke, and increased susceptibility to infections [2–4]. Sub-Saharan Africa bears the highest burden of this disease, with Nigeria accounting for nearly 150,000 births annually [5]. Despite advances in the understanding and management of SCA, children in resource-limited settings continue to experience high morbidity and mortality due to inadequate healthcare infrastructure and limited access to effective therapies [6–8].

Hydroxyurea (also known as hydroxycarbamide) is an oral medication that has become a cornerstone of SCA management. Hydroxyurea increases foetal haemoglobin production, which in turn inhibits the polymerization of sickle haemoglobin and reduces red cell sickling [9]. Numerous clinical trials and observational studies have demonstrated that hydroxyurea therapy significantly decreases the frequency of painful crises, hospitalizations, and the need for blood transfusions in SCA patients [10, 11]. Its efficacy in improving quality of life and overall survival has led to recommendations for its use as a standard of care for SCA, including endorsement by the World Health Organization (WHO) [12, 13].

However, the uptake of hydroxyurea in low- and middle-income countries (LMICs) has been limited by multiple challenges [14]. Key barriers include the high cost of the medication, the requirement for regular laboratory monitoring, and persistent misconceptions or lack of knowledge about the drug’s safety among some healthcare providers and caregivers [14–16]. In sub-Saharan Africa, these issues are compounded by constrained healthcare resources and socio-economic difficulties faced by families [17–20]. Moreover, treatment adherence remains a significant concern in chronic diseases like SCA, particularly in resource-limited settings where factors such as medication cost, complex dosing regimens, and inadequate patient education can negatively impact compliance [17, 20–22].

To address these challenges, our tertiary hospital in Southeast Nigeria implemented a simplified dosing regimen for hydroxyurea for paediatric patients with sickle cell anaemia. The goal of this simplified dosing strategy was to balance the proven clinical benefits of hydroxyurea with the practical constraints of a resource-limited setting. By administering a uniform regimen (20 mg/kg per day, capped at 500 mg for older children above a certain weight), we hypothesized that we could achieve meaningful reductions in SCA complications while minimizing the need for frequent laboratory tests. This simplification was expected to reduce the financial burden on families and improve treatment adherence through an easier, one-size-fits-all dosing approach [19].

This study reports our experience with this treatment strategy over two years, focusing on its impact on emergency clinic presentations, treatment adherence, and overall patient outcomes. We hope that our findings will contribute to the growing body of evidence supporting the use of fixed dose hydroxyurea in the management of SCA in resource-limited settings and encourage further research to optimize treatment protocols and adherence strategies for this vulnerable population.

## Methodology

### Study design and setting

This was a prospective open-label cohort study conducted over a two-year period (January 2022 through January 2024) at Nnamdi Azikiwe University Teaching Hospital (NAUTH) in Nnewi, Anambra State, Nigeria. NAUTH is a tertiary referral centre in South-Eastern Nigeria with a dedicated paediatric haematology clinic that manages a large number of children with SCA.

### Study population and recruitment

All eligible children presenting to the sickle cell clinic during the study period were approached for participation. Of 102 children approached, 2 declined participation. One hundred children were ultimately enrolled, commenced hydroxyurea, and assessed for outcomes at 24 months.

**Fig. 1:**
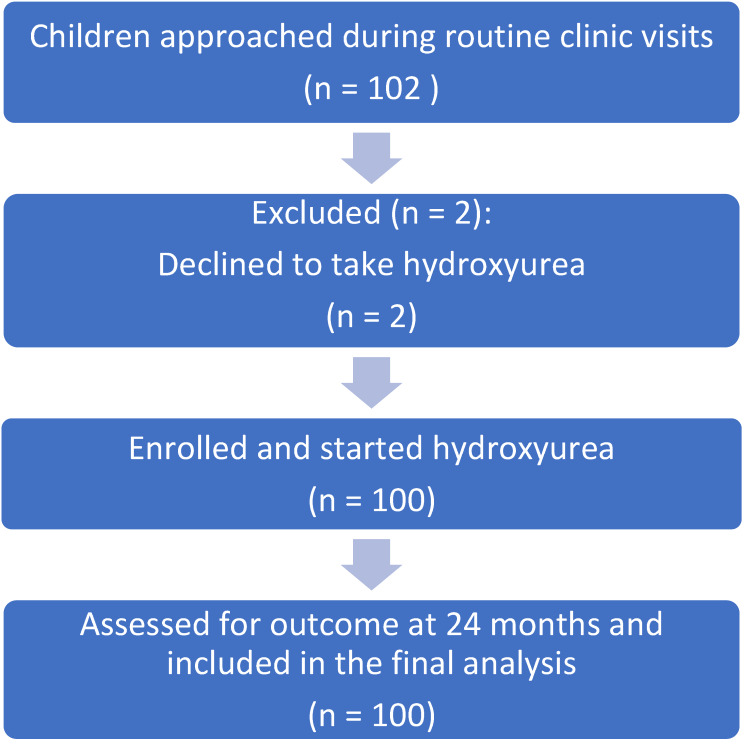
CONSORT-style flow diagram showing participant enrolment, treatment, and follow-up over 24 months

A CONSORT-style flow diagram (Fig. 1) summarises this process.

#### Inclusion criteria

Children aged 1–18 years with a confirmed diagnosis of SCA (HbSS genotype, verified by haemoglobin electrophoresis) who were regular attendees of the paediatric haematology clinic at NAUTH were eligible for inclusion. We included both male and female patients within this age range, provided they had not previously been on hydroxyurea therapy.

#### Exclusion criteria

We excluded children who had received hydroxyurea prior to the study and those with any known contraindications to hydroxyurea (such as significant baseline cytopenia or hepatic/renal dysfunction that would preclude safe use of the drug).

Patients were prescribed hydroxyurea and followed passively over time, with outcomes monitored through emergency room presentations documented in hospital records. Due to irregular follow-up patterns common in our setting, no participant was excluded solely for missing clinic visits [23].

Eligible patients (meeting the above criteria) were consecutively enrolled during their routine clinic visits. In total, 100 children were enrolled for the study. After obtaining informed consent, each patient was initiated on the simplified hydroxyurea dosing regimen and followed prospectively.

Although no formal laboratory screening was performed due to financial and logistical constraints, all participants were clinically assessed for evidence of pallor, active infection, or signs of organ dysfunction. Only children deemed clinically stable were commenced on hydroxyurea. This pragmatic approach reflects common practice in similar low-resource settings, where full laboratory evaluation may not be feasible.

### Treatment protocol

At the time of this study, hydroxyurea was not routinely prescribed in our centre, and this represented the first structured use of the medication for paediatric sickle cell anaemia at our institution. The drug was sourced through the hospital pharmacy and procured directly by caregivers. It was not provided by the study, and families bore the full cost of the medication.

The dosing regimen adopted in this study differed from conventional practice, which typically involves initiating hydroxyurea at 10–15 mg/kg/day with dose escalation based on regular full blood count (FBC) monitoring to reach the maximum tolerated dose (MTD). However, in our setting, this approach is often impractical due to the financial burden associated with serial laboratory investigations and medication procurement. Consequently, a simplified fixed-dose strategy was selected as a pragmatic alternative to improve access, minimise monitoring requirements, and support long-term adherence in a resource-constrained environment.

All enrolled patients were initiated on hydroxyurea at 20 mg/kg/day administered orally. To prevent overdosing in heavier or older children, a maximum dose cap of 500 mg/day was applied. Thus, children weighing more than 25 kg received a fixed daily dose of 500 mg.

Hydroxyurea was dispensed in 500 mg capsules. For children weighing less than 25 kg, the total weekly dose was calculated by multiplying body weight (kg) by 20 mg/kg/day and then by 7 days. This total weekly dose was then divided by 500 mg to determine the number of capsules required per week. Based on this calculation, children received 500 mg on the appropriate number of days per week and skipped the remaining days. For example, a child weighing 18 kg (total weekly dose 2520 mg) received one 500 mg capsule daily for five days each week. Dosing schedules were adjusted during follow-up visits if weight gain was substantial. This approach allowed for safe and consistent dosing despite the unavailability of lower-dose formulations or paediatric suspensions. For children unable to swallow capsules, prescriptions were compounded into liquid formulations by the hospital pharmacy to ensure accurate dosing and avoid potential drug loss from manual capsule opening.

Patients were evaluated monthly for follow-up and clinical monitoring of signs suggestive of toxicity. In the event of a “breakthrough” vaso-occlusive crisis—defined as a pain episode severe enough to warrant an emergency department visit—dose escalation was implemented per protocol. For such patients, the dose was increased to 25 mg/kg/day, capped at 750 mg/day. Following dose adjustments, patients were closely observed for clinical response and potential side effects.

Although routine laboratory monitoring was not feasible due to financial constraints, clinical assessments were conducted monthly, and any signs suggestive of toxicity (e.g., bruising, infections, fatigue) prompted targeted laboratory testing. No cases of clinically significant haematologic toxicity requiring treatment discontinuation were observed.

### Data collection

Data were collected using standardized case report forms at baseline and during follow-up visits. Baseline data included demographic information (age, sex), anthropometric measurements (weight, height, from which body mass index [BMI] was calculated), and socio-economic status (categorized into lower, middle, and upper social class based on parental occupation and education, according to local standard classifications). We also recorded the family history details such as parental ages, since these were later examined for associations with adherence.

Clinical outcomes of interest were defined prior to the study. The primary outcome was the number of SCA-related emergency room (ER) visits during the 24-month study period. Each ER visit for a vaso-occlusive pain crisis or other SCA complication was counted. The secondary outcome was treatment adherence to the hydroxyurea regimen.

We also monitored safety outcomes by documenting any adverse events reported by caregivers or observed during clinic visits. Particular attention was paid to symptoms potentially indicative of hydroxyurea toxicity (such as frequent infections, bleeding, or gastrointestinal disturbances). However, comprehensive lab monitoring for toxicity was limited, so safety assessment largely relied on clinical observation.

#### Adherence assessment

Adherence was evaluated at each monthly visit using caregiver and patient self-reports. Caregivers (or older patients, when applicable) were asked about missed doses of hydroxyurea within the preceding two-week period. “Poor adherence” was defined as missing three or more doses during this timeframe, corresponding to less than approximately 80% adherence. Participants who missed fewer than three doses were classified as adherent. This binary classification was used to estimate overall adherence and to explore associated demographic and clinical factors.

This method has been employed in other paediatric studies in sub-Saharan Africa, including the REACH trial [19], where caregiver-reported adherence served as a practical alternative in the absence of electronic tracking tools. While self-report may overestimate true adherence, it remains a feasible and context-appropriate proxy in resource-constrained settings.

### Data analysis

All data were entered into an electronic spreadsheet and analysed using Python (version 3.11.5). Descriptive statistics were used to summarize patient characteristics and outcomes. We calculated means and standard deviations for approximately normally distributed continuous variables (e.g., age, BMI) and medians with ranges (or interquartile ranges) for skewed variables (e.g., parental ages, which were not strictly normally distributed). Categorical variables (such as sex, social class, adherence status) were summarized as frequencies and percentages.

To address the study objectives, we performed comparative analyses between subgroups where relevant. In particular, we compared patients who were adherent to those who were non-adherent to identify factors associated with adherence. For continuous variables like child age and parental ages, we used Student’s t-test for independent samples if the data were normally distributed. In cases of non-normal distribution (as was the case for father’s age), we used the non-parametric Mann–Whitney U test to compare medians. We compared categorical variables (such as sex, social class, and age-category groups) between adherence groups using the chi-square test. Fisher’s exact test was applied in place of chi-square if an expected cell count in the contingency table was < 5.

The effect of age on adherence was further explored by categorizing age into groups (1–5, 6–10, 11–15, and > 15 years) and comparing adherence rates across these groups. Trends in adherence by age category were assessed using chi-square tests for trend.

A two-tailed p-value of < 0.05 was considered statistically significant for all analyses. The primary outcome (ER visit frequency) was described in proportion terms and was too low for meaningful statistical comparison within the cohort (since almost no events occurred). No imputation was needed for missing data, as key variables were fully observed for all participants (any patient who was lost to follow-up was not included in the final analysis set).

All results are presented with appropriate measures of uncertainty (± standard deviation for means, or with specified p-values for comparisons).

## Results

### Patient demographics

A total of 100 children with SCA were included in the study analysis (after excluding a few who were lost to follow-up). The median age of participants at enrolment was 9.0 years (range: 1–18 years), with an almost even distribution across different paediatric age categories. 58% of the patients were male (male: female ratio approximately 1.4:1). A substantial proportion of participants (85%) adhered to the fixed moderate-dose hydroxyurea regimen. Table 1 summarizes the baseline demographic and clinical characteristics of the study cohort.

**Table 1:** Demographic and clinical characteristics of study participants

Variables	Average/Frequency (*n*)	Range/Percentage (%)
Median Age (years)	9.0 (5.0–14.0)	7 months-18.0
Age Category (years)		
Below 5	32	32.0
6–10	26	26.0
11–15	27	27.0
Above 15	15	15.0
Gender		
Male	58	58.0
Female	42	42.0
Median Weight (kg)	26.0 (17.0-38.57)	5.0–70.0
Median Height (cm)	127.7 ± 28.4	61.0-186.0
Median BMI (kg/m²)	15.4 (14.4–16.6)	11.8–24.8
Mother Age (years)	40.0 (35.0–50.0)	25.0–55.0
Father Age (years)	44.0 (40.0-50.7)	27.0–79.0
Social Class		
Upper	34	34.0
Middle	38	38.0
Lower	28	28.0
Fixed Moderate-Dose Hydroxyurea Adherence		
Yes	85	85.0
No	15	15.0

### Clinical outcomes

#### Emergency room visits (primary outcome)

Over the two-year follow-up, the incidence of SCA-related emergency room visits was extremely low in our treated cohort. Only one patient out of 100 (1%) required an emergency visit for a vaso-occlusive pain crisis while on the fixed-dose hydroxyurea therapy. This single event is noteworthy given that historically, children with SCA in this context would be expected to have multiple crises requiring urgent care. The patient who experienced the breakthrough vaso-occlusive crisis was an older adolescent who had been on the maximum fixed dose of 500 mg/day. In accordance with our protocol, after this ER visit the patient’s hydroxyurea dose was escalated to 750 mg/day (approximately 25 mg/kg for that patient). Following the dose increase, no further emergency visits or severe crises were recorded for this individual in the remaining study period. Thus, 99 out of 100 patients (99%) had **zero** SCA-related ER visits during 24 months of therapy.

#### Hydroxyurea dose adjustments

Of the 100 patients, 99 patients (99%) maintained the initial fixed moderate-dose regimen throughout the study period. 1 patient (1%) required a dose increase due to a breakthrough crisis. The patient who experienced the breakthrough crisis was initially on the capped dose of 500 mg/day. Following the crisis, their dose was increased to 750 mg/day as per the study protocol. No further emergency presentations were recorded for this patient after the dose adjustment.

#### Safety and tolerability

No significant clinical side effects were observed during the study period. The fixed moderate-dose regimen was well-tolerated by all patients based on caregiver reports and clinical assessments during follow-up visits.

### Treatment adherence and associated factors

#### Overall adherence rate

Out of the 100 children, 85 (85%) were classified as adherent to the hydroxyurea regimen throughout the study. These patients (or their caregivers) reported taking the medication consistently, with minimal missed doses. On the other hand, 15 patients (15%) were non-adherent, meaning they frequently missed doses (3 or more doses in a two-week interval on multiple occasions).

#### Factors associated with fixed moderate-dose hydroxyurea adherence

The comparison of patient characteristics by hydroxyurea compliance status revealed significant associations with age and father’s age. Patients who were non-compliant with hydroxyurea therapy had a significantly higher mean age (11.6 ± 4.2 years) compared to those who were compliant (8.5 ± 5.3 years), with a p-value of 0.027, indicating that younger patients are more likely to adhere to the treatment. Additionally, the age of fathers showed a significant difference, with non-compliant patients having older fathers (median age 49.0 years) compared to compliant patients (median age 44.0 years), with a p-value of 0.02. Other variables such as gender, BMI, mother’s age, social class, and age categories did not show significant associations with hydroxyurea compliance (Table 2).

**Table 2:** Comparison of patient characteristics by hydroxyurea adherence status

	Not Adherent (*n* = 15)	Adherent (*n* = 85)	*p*-value
Age (years)	11.6 ± 4.2	8.5 ± 5.3	0.027**
Age Category (years)			
Below 5	2 (13.3)	30 (35.3)	
6–10	4 (26.7)	22 (25.9)	0.242
11–15	6 (40.0)	22 (25.9)	
Above 15	3 (20.0)	11 (12.9)	
Gender			
Female	7 (46.7)	35 (41.2)	0.999
Male	8 (53.3)	50 (58.8)	
BMI (kg/m²)	16.5 ± 2.1	15.7 ± 2.5	0.243
Mother Age (years)	43.3 ± 10.9	65.4 ± 21.0	0.677
Father Age (years)	49.0 (45.5-54.3)	44.0 (38.0-50.0)	0.020**
Social Class			
Lower	5 (33.3)	23 (27.1)	
Middle	5 (33.3)	34 (40.0)	0.804
Upper	5 (33.3)	28 (32.9)	

## Discussion

This study provides valuable insights into the use of a simplified hydroxyurea dosing regimen for children with SCA in a resource-limited African setting. Over a two-year period, our findings suggest that this dosing approach may be associated with a significant reduction in acute SCA complications and appears to support high treatment adherence, without compromising safety.

We recorded only one emergency room visit for a vaso-occlusive crisis among 100 participants over a two-year period, reflecting an exceptionally low event rate. This outcome highlights the potential role of this simplified hydroxyurea dosing strategy in transforming SCA care in low-resource settings, where frequent hospitalizations and poor adherence remain major challenges [1, 24]. Our findings align with and extend observations from other studies on hydroxyurea use in Africa. The multicentre REACH trial in sub-Saharan Africa, for example, utilized a fixed dose of ~ 17.5 mg/kg/day of hydroxyurea in children and reported substantial clinical benefits, including reductions in painful crises and acute chest syndrome events [17, 19]. Those results demonstrated that even without maximal dose escalation, African children experienced fewer SCA complications on hydroxyurea, supporting the practicality of a fixed-dose approach. Similarly, our regimen of 20 mg/kg/day (with a 500 mg cap) appeared effective. The fact that we needed to escalate the dose in only one patient reinforces the idea that many children can be well-managed without complex titration.

Current dose-escalation protocols for hydroxyurea aim to reach the maximum tolerated dose for optimal clinical effect [10]. However, they require frequent laboratory monitoring and specialised expertise, which are often not available in many low-resource settings [19, 25]. In contrast, our simplified dosing regimen provides a more accessible alternative that can be implemented more broadly without the need for intensive infrastructure. Although a capped dosing strategy may theoretically result in under-treatment for older or heavier children, our outcomes, particularly the low rate of emergency room visits, suggest that clinical benefit was preserved. Furthermore, none of the patients assessed during routine clinical evaluations showed signs of drug-related toxicity, supporting the safety and practicality of this approach in resource-constrained environments.

One important observation from our study is the high adherence rate observed of 85%. This is particularly encouraging in a resource-limited setting, where treatment compliance is often difficult to sustain [17, 20]. The simplified dosing regimen and reduced need for frequent monitoring may have contributed to this high adherence rate. Similarly, our approach of capping the dose at 500 mg/day for older children which aimed to balance efficacy with safety, considering the financial constraints and limited healthcare infrastructure typical of low- and middle-income countries (LMICs) may have further promoted adherence [19].

It is also worth discussing the age-related adherence pattern that was observed. Younger children had better adherence than adolescents. This trend is understandable: younger children’s treatment is entirely managed by parents, whereas adolescents may begin to take on responsibility or may occasionally resist taking medication as part of asserting independence. Moreover, teenagers with chronic illness often face psychosocial challenges that can interfere with adherence such as feeling “different” from peers, or simple fatigue from years of daily medicine [26]. Our data underscore the need for targeted strategies to support adolescents with SCA on hydroxyurea. This could include interventions like reminder apps, peer support groups, or more frequent counselling tailored to older children [27]. It might also involve actively engaging the adolescent in understanding their condition and the importance of the medication to encourage self-motivation. Since SCA complications can be especially dangerous in adolescence and early adulthood, maintaining high adherence in that age group is critical. Future programs might consider transition clinics or special adherence incentives for teenagers to bridge this gap.

The association between paternal age and hydroxyurea adherence observed in this study suggests that family dynamics play a crucial role in chronic disease management in children with sickle cell anaemia (SCA) [28]. Older fathers may have less direct involvement in daily caregiving, potentially leading to greater reliance on other family members, which could influence medication consistency. Additionally, older paternal age may correlate with larger household sizes or caregiving by grandparents, both of which could impact treatment routines and adherence [27]. Given the complexity of adherence in paediatric SCA management, further research is needed to examine how family composition and caregiver roles influence treatment outcomes. Future interventions should adopt family-centered education strategies to ensure that all caregivers—including fathers and non-primary guardians—are actively engaged in supporting medication adherence and optimizing long-term treatment outcomes [28, 29].

In terms of safety, the lack of routine laboratory monitoring is often cited as a barrier to hydroxyurea use in LMICs. However, our experience showed no clinically evident haematologic toxicity over two years, even with limited lab testing. This supports the notion that at modest doses, hydroxyurea is generally well tolerated. This aligns with evidence that at doses around 15–20 mg/kg, hydroxyurea rarely causes severe neutropenia if patients are reasonably healthy at baseline [30, 31]. We do acknowledge that monitoring is still important and we do not advocate ignoring safety labs entirely but in scenarios where lab facilities are sparse with poor follow-up rates [23], a compromise approach might be to perform periodic checks every few months, or when clinically indicated, rather than foregoing hydroxyurea altogether. Moreover, point-of-care devices for hemograms or emerging low-cost testing methods could be introduced to facilitate basic monitoring even in remote areas. The safety profile we observed (no obvious drug-related complications) should reassure health providers that a simplified dosing regimen is feasible and likely to be safe. Future research should aim to incorporate cost-effective monitoring solutions to ensure long-term safety while maintaining the practicality of the fixed moderate-dose approach.

This study offers several important strengths. It provides real-world evidence from a vulnerable paediatric population in a resource-constrained African setting, an area where data on hydroxyurea use remain scarce. This simplified hydroxyurea dose regimen was not only feasible and safe but also achieved notably high adherence rates over a two-year period, despite limited access to routine laboratory monitoring. The study further contributes practical insights by identifying adherence patterns across age groups and highlighting how caregiver dynamics, such as paternal age, may influence treatment consistency. These findings strengthen the case for adopting a pragmatic, fixed-dose approach to hydroxyurea in similar low-resource environments. While this study offers valuable insights, there are important limitations to acknowledge. The absence of a systematically recorded emergency room data prior to hydroxyurea initiation limits the ability to attribute outcomes solely to the intervention. Adherence was assessed through caregiver self-report, which may introduce recall or social desirability bias. Laboratory monitoring was limited due to resource constraints, which may have reduced our ability to detect subclinical toxicity. Importantly, the primary outcome focused on emergency room visits, and while pain episodes managed at home may have occurred, they were not captured because they fell outside the defined scope of our study. This approach, however, reflects real-world care in our setting, where ER visits are a practical and observable marker of disease severity. The consistency of our findings and the clinical impact observed suggest that this simplified hydroxyurea dosing protocol holds substantial promise for similar contexts.

In conclusion, this prospective cohort study suggests that a simplified hydroxyurea dosing regimen may be a feasible and context-appropriate approach for managing paediatric sickle cell anaemia in resource-limited settings. We observed a marked reduction in serious SCA-related complications and high levels of treatment adherence, which may reflect the practicality and acceptability of the simplified protocol. This approach has the potential to reduce the clinical burden of SCA by decreasing emergency room visits and supporting better day-to-day health, even in the absence of comprehensive laboratory monitoring infrastructure.

We recommend that healthcare providers and policy-makers in high-burden regions explore the integration of this simplified hydroxyurea dosing protocol into routine care, while ensuring that supportive measures such as caregiver education and adherence reinforcement are put in place. With further validation, this model could contribute meaningfully to the transformation of SCA care in low-resource environments, enabling more children to lead healthier, crisis-free lives. As global health strategies increasingly prioritise non-communicable diseases in LMICs, paediatric sickle cell anaemia should receive commensurate focus, and this study underscores how pragmatic, evidence-informed solutions can be both effective and scalable.

## Electronic supplementary material

Below is the link to the electronic supplementary material.

Supplementary Material 1

## Data Availability

The dataset supporting the conclusions of this article has been uploaded as a supplementary file and is available with the published manuscript.

## Abbreviations

SCA: Sickle cell anaemia
LMICs: Low- and middle-income countries
FBC: Full blood count
ER: Emergency room
